# Cancer-associated fibroblasts promote migration and invasion of non-small cell lung cancer cells via METTL3-mediated RAC3 m^6^A modification

**DOI:** 10.7150/ijbs.79467

**Published:** 2023-03-05

**Authors:** Mengmeng Chen, Qicheng Zhang, Sijia Zheng, Xueru Guo, Limin Cao, Yinghui Ren, Yongmei Qian, Min Wang, Xiang Wu, Ke Xu

**Affiliations:** 1 Tianjin Key Laboratory of Lung Cancer Metastasis and Tumor Microenvironment, Tianjin Lung Cancer Institute, Tianjin Medical University General Hospital, Tianjin 300052, China.; 2 Department of Clinical Laboratory, Shandong Cancer Hospital and Institute, Shandong First Medical University and Shandong Academy of Medical Sciences, Jinan 250117, China.; 3 Department of Anesthesiology, Tianjin First Central Hospital, Tianjin 300192, China.; 4 Core Facility Center, Tianjin Medical University General Hospital, Tianjin 300052, China.

**Keywords:** Lung cancer, Metastasis, Cancer-associated fibroblasts, m^6^A, METTL3, RAC3

## Abstract

Cancer progression depends on the communication between tumor cells and tumor microenvironment. Cancer-associated fibroblasts (CAFs) are a major component of stromal cells. CAFs promote cancer metastasis; however, it has not been evaluated whether N6-methyladenosine (m^6^A) modification is responsible for CAFs' role in metastasis. In the present study, we found that CAFs promoted migration and invasion of non-small cell lung cancer (NSCLC) cells by elevating m^6^A modification in NSCLC cells. Methyltransferase-like 3 (METTL3) in NSCLC cells mediated CAFs' effect on m^6^A modification, and was regulated by CAFs-secreted vascular endothelial growth factor A (VEGFA). METTL3 knockdown in NSCLC cells dramatically inhibited cell migration and invasion, and suppressed tumor growth *in vivo*. Database analysis revealed that METTL3 was associated with poor prognosis of lung cancer. The mechanism study showed that METTL3 increased m^6^A level of RAC3 mRNA, resulting in increased stability and translation of RAC3 mRNA. RAC3 was responsible for the CAFs' promoting effect on cell migration via the AKT/NF-κB pathway. This study established a CAF-METTL3-RAC3 m^6^A modification-dependent regulation system in NSCLC metastasis, suggesting potential candidates for metastasis treatment.

## Introduction

Lung cancer is the most lethal cancer worldwide, with nearly 1.8 million new deaths (18.0% of all cancer deaths) reported in 2020 1. The major pathological type of lung cancer is NSCLC, which represents 85% of lung cancer cases. NSCLC mainly comprises lung adenocarcinoma (LUAD), lung squamous cell carcinoma (LUSC), and large cell carcinoma 2. Despite treatment advances in recent years, the mortality rate of lung cancer remains high. Metastasis is the main cause of lung cancer-related death as it is responsible for 90% of patients' deaths 3; therefore, an in-depth understanding of the mechanism underlying lung cancer metastasis is urgently needed.

Tumor microenvironment (TME) plays a crucial role in tumorigenesis and tumor progression. The interaction between tumor cells and TME facilitates tumor growth by regulating immune escape, angiogenesis, inflammation, drug response, and metastasis; it also remodels TME to be favorable for tumor growth 4. TME comprises different elements, such as tumor cells, stromal cells, immune cells, cytokines, extracellular matrix, and blood vascular network. CAFs are major stromal cells in TME, and they stimulate tumor progression via secretion of cytokines and exosomes. CAF-derived CCL5 enhances metastasis of hepatocellular carcinoma through the activation of the HIF1α/ZEB1 axis 5. CAF-released interleukin-8 promotes stemness and malignancy of ovarian cancer cell by activating the Notch3 pathway 6. Exosomal miR-18b derived from CAFs stimulates breast cancer metastasis by regulating TCEAL7 7. Our previous studies also revealed that CAFs promoted metastasis of lung cancer cells via the secretion of KRT8 and HMGB1 8, 9.

The epigenetic modifications, including DNA methylation and RNA methylation, have emerged as crucial regulators of diverse physiological and pathological processes. Among various mRNA methylations, m^6^A is the most abundant RNA modification, and there are nearly 3-5 m^6^A modifications in each mRNA. m^6^A methylation mainly occurs in the consensus sequence RRACH (R = A or G; H = A, C, or U) 10. It is catalyzed by the methyltransferase complex (MTC) (termed as “writers”) and demethylase (termed as “erasers”), and recognized by “readers.” m^6^A methylation plays an important role in RNA fate through influencing pri-mRNA splicing and RNA decay, export, and translation 11.

Recent studies have revealed that dysregulated m^6^A modification is involved in disease progression. m^6^A modification plays an important role in the development of osteoarthritis (OA) 12; contributes to the pathophysiology of cardiovascular diseases by regulating cell proliferation, differentiation, autophagy, and inflammation 13; and is responsible for hippocampal memory deficits in Huntington disease 14. In particular, m^6^A modification contributes to cancer progression. METTL3 promotes carcinogenesis of renal cell carcinoma by regulating HHLA2 m^6^A modification 15, while demethylase ALKBH5 inhibits gastric cancer cell invasion by regulating PKMYT1 m^6^A modification 16. Yao B *et al.* reported that colorectal cancer progression was stimulated through m^6^A-mediated stabilization of CREB1 mRNA 17.

Different mechanisms underlying CAFs' effect on lung cancer progression have been revealed; however, it has not been evaluated whether m^6^A methylation is involved. In this study, we found that CAFs promoted the metastatic potential of NSCLC cells by regulating m^6^A methylation in NSCLC cells, and demonstrated that METTL3 mediated the CAFs' effect on m^6^A methylation. We further identified RAC3 as a modified target of METTL3, and RAC3 stimulated NSCLC cells migration through AKT/NF-κB signaling.

## Materials and methods

### Reagents and antibodies

Recombinant human VEGFA and VEGFA-neutralizing antibody were purchased from R&D Systems (Minneapolis, MN). Antibodies against METTL3 (#86132), FTO (#31687), AKT (#4685), p-AKT (#4060), and p65 (#8242) (in 1:1000 dilution) were purchased from Cell Signaling Technology (Beverly, MA); anti-ALKBH5 (# ab69325, 1:1000 dilution) was purchased from Abcam (Cambridge, UK); anti-m^6^A (#202003, 1:1000 dilution) was purchased from Synaptic Systems (Göttingen, Germany); anti-RAC3 (#16117-1, 1:500 dilution) was purchased from Proteintech (Rosemont, IL); anti-p-p65 (#WL02169), anti-MMP-9 (#WL03096), and anti-TWIST1 (#WL00997) (in 1:1000 dilution) were purchased from Wanleibio (Shenyang, China); anti-β-actin (#ab8224, 1:4000 dilution) was purchased from Sigma-Aldrich (St. Louis, MO); and goat anti-rabbit (#ZB-2301, 1:8000 dilution) and anti-mouse IgG (#ZB2305, 1:8000 dilution) were purchased from ZSGB-BIO (Beijing, China).

### Cell culture and lung stromal fibroblasts

NSCLC cell lines (A549, H1299, and H661) were purchased from American Type Culture Collection (Manassas, VA). A549 cells were cultured in DMEM medium, and H1299 and H661 cells were cultured in RPMI-1640 medium, with 10% fetal bovine serum (GIBCO; Grand Island, NY). The cells were cultured at 37°C and 5% CO_2_.

Fibroblasts were isolated from tumor and adjacent non-tumor tissues of NSCLC patients who underwent surgery at Tianjin Medical University General Hospital (TMUGH, Tianjin, China), and CAF conditioned medium (CAF-CM) was collected as previously described 9. The informed consent was gained from the patients. The study received the permission from the institutional Ethical Review Committee (Registration number IRB2020-KY-224).

### Cell proliferation assay

NSCLC cells were plated on 96-well plates at a density of 5×10^3^ cells/well. The cells were grown for 48 h after treatments, and cell proliferation was measured by CCK-8 kit (Dojindo, Kumamoto, Japan) in accordance with the manufacturer's instruction. The absorbance at A450 was measured on a microplate reader (BioTek, Santa Clara, CA).

### Colony formation assay

NSCLC cells were plated on 12-well plates with 500 cells/well. The cells were cultured successively for 10-14 days. Colonies were stained by 1% crystal violet. Images were scanned by a scanner (Canon, Tokyo, Japan), and the colonies were counted.

### Cell migration assay

The ability of cell migration was assessed by the wound healing assay as previously described 18. Cells were plated on 6-well plates, pretreated with 0.05 μM mitomycin C, and cultured under different conditions. A pipette tip was used to make a linear scratch. Pictures were taken by a microscope (Nikon, Tokyo, Japan) after 24 h to evaluate cell migration by the wound closure.

### Cell invasion assay

Cell invasion was assessed by the transwell assay as previously described 8. Briefly, the chambers were coated with 40 μL Matrigel (BD Biosciences, CA) and put in 24-well plates. Cells (1×10^5^) were pretreated with 0.05 μM mitomycin C, suspended in serum-free medium, and seeded into the chamber. The conditioned medium was added to the lower area of the chamber. After 48 h of culture, the invaded cells were stained using 1% crystal violet. The images were taken by a microscope (Nikon, Tokyo, Japan), and the invaded cells were counted.

### Enzyme-linked immunosorbent assay (ELISA)

VEGFA levels in supernatants were measured by ELISA as previously described 8. Briefly, the supernatants were collected, and VEGFA levels were measured by human VEGFA ELISA kit in accordance with the kit instruction (RayBiotech Life, Peachtree Corners, GA). The absorbance at A450 was measured on a microplate reader (BioTek, Santa Clara, CA).

### RNA interference and plasmid transfection

NSCLC cells were transfected with siMETTL3, siRAC3, control siRNA (GenePharma, Shanghai, China), or pMETTL3, pRAC3, and control vector (Public Protein/Plasmid Library, Nanjing, China) using Lipofectamine 3000 (Invitrogen, Carlsbad, CA) following the manufacturer's instructions. The sequences of siRNA duplex were as follows: for METTL3, sense 5'-GCAAGAAUUCUGUGACUAUTT-3', antisense 5'-AUAGUCACAGAAUUCUUGCTT-3'; for RAC3, sense 5'- GCAGCUGAAAUAUCCUAAATT-3', antisense 5'-UUUAGGAUAUUUCAGCUGCTT-3'; for control, sense 5'-UUCUCCGAACGUGUCACGUTT-3', antisense 5'-ACGUGACACGUUCGGAGAATT-3'.

### RNA stability assay

NSCLC cells were plated on 12-well plates. Actinomycin D (5 μg/mL) was added to the plates. The cells were grown for 4-8 h. RNA was extracted using TRIzol (Invitrogen, Waltham, MA), and the amounts of RNA were quantified on a Nanodrop 2000 (ThermoFisher Scientific, St. Louis, MO). The mRNA levels were detected by quantitative polymerase chain reaction (qPCR).

### Western blotting

Cells were lysed in RIPA buffer (Beyotime, Shanghai, China) containing protease inhibitor (Sigma-Aldrich). Protein was quantified using Pierce BCA Protein Assay kit (Thermo Fisher Scientific). Proteins were separated by 12% SDS-PAGE and transferred onto nitrocellulose membranes (Millipore, St. Louis, MO). After blocking with *Tris-buffered saline* (TBS) containing 5% milk and 0.5% Tween-20 for 1 h, the membranes were probed with primary antibodies and the corresponding HRP-conjugated secondary antibodies. The blots were visualized using an ECL system (Thermo Fisher Scientific).

### qPCR

qPCR was conducted as previously described 19. RNA was extracted from cells using TRIzol (Invitrogen), and reverse transcription was carried out using the Takara kit (Dalian, China). Gene expression was assessed by qPCR using the Power SYBR-Green Master Mix (ThermoFisher Scientific). The primer sequences are listed in Table 1. GAPDH was used for normalization.

### RNA m^6^A methylation assay

The m^6^A modification levels of mRNA were measured by the EpiQuik m^6^A RNA Methylation Quantification Kit (Epigentek, Farmingdale, NY), following the manufacturer' instruction. Briefly, 200 ng RNA were added to assay wells, and the capture antibody was added to the assay wells. After incubation, the A450 was measured, and the levels of m^6^A modification were calculated according to the standard curve.

### RNA m^6^A dot blots

Total RNAs were denatured at 65°C for 5 min. RNA (300 ng) was spotted onto a nylon membrane (Beyotime). The membranes were UV-crosslinked for 10 min, then blocked with 5% milk, and incubated with anti-m^6^A antibody. After washing with TBS-T, the membranes were probed with the corresponding HRP-conjugated secondary antibodies. The blots were visualized using the ECL system (Thermo Fisher Scientific). The membranes were also stained with 0.02% methylene blue (Sangon Biotech, Shanghai, China) in 0.3 M sodium acetate. Methylene blue staining was used for RNA loading control.

### Methylated RNA immunoprecipitation sequencing (MeRIP-seq)

MeRIP-seq was performed as previously reported, with minor modifications 20. Briefly, RNA was extracted and purified using RNeasy MinElute Cleanup Kit (Qiagen, Hilden, Germany). RNA was sheared into approximately 100-nt fragments and incubated with anti-m^6^A antibody. Protein A/G magnetic beads (Thermo Fisher Scientific) in immunoprecipitation buffer were added and incubated at 4°C overnight. Antibody-bound methylated RNA was eluted and purified for further MeRIP-seq by LC-Bio Technology (Hangzhou, China). RNA sequencing was also conducted by LC-Bio Technology.

### Methylated RNA immunoprecipitation quantitative PCR (MeRIP-qPCR)

MeRIP-qPCR assay was performed in accordance with a previous report 20. Briefly, RNA was purified, and 1/10 of RNA was used for input control. Protein A/G magnetic beads were incubated with anti-m^6^A antibody or rabbit IgG at 4°C for 2 h. After washes, the antibody-conjugated beads were mixed with RNA in immunoprecipitation buffer containing RNase inhibitors. The methylated mRNAs were precipitated, and the enrichment was assessed by qPCR. The m^6^A enrichment was calculated via input normalization.

### RNA immunoprecipitation (RIP) assay

RIP assays were performed using the Magna RIP RNA-Binding Protein Immunoprecipitation Kit (Millipore, St. Louis, MO). Briefly, protein A/G magnetic beads coated with antibodies against rabbit immunoglobulin G or METTL3 were incubated with cell lysates at 4°C overnight. After washes, proteinase K digestion buffer was added to the RNA-protein mixture. Finally, RNAs were extracted by TRIzol. The interaction between METTL3 and RAC3 was determined by qPCR.

### Xenograft mouse model

The xenograft study received the permission from the institutional Ethical Review Committee (Registration number ZYY-DWFL-IRB- 002(F)-01). Nude mice were assigned randomly into two groups. For the first group, 2.5×10^6^ A549 cells were injected subcutaneously into the right flank of mice, and 2.5×10^6^ A549 cells mixed with 5×10^6^ CAFs were injected into the left flank. For the second group, 2.5×10^6^ A549-METTL3-KD cells were injected into the right flank, and A549-METTL3-KD cells mixed with 5×10^6^ CAFs were injected into the left flank. Tumor volumes were measured every week. The tumor volume was calculated as follows: Volume = *d^2^ × D/2*, where* D* is the longest diameter and *d* is the shortest diameter. The experiment was terminated after 5 weeks, and tumor tissues were removed for further examinations.

### Statistical analysis

All data were presented as mean ± SD and were from more than three independent experiments. The statistical analysis was performed using GraphPad Prism 9 (GraphPad Software, San Diego, CA). The differences between two groups were assessed by two-tailed Student's *t*-tests, and the differences among different groups were analyzed by one-way ANOVA. P < 0.05 was considered to be statistically significant.

## Results

### CAFs enhance the growth, migration, and invasion of NSCLC cells

To investigate the effect of CAFs on NSCLC cells, CAFs and normal fibroblasts (NFs) were collected from lung cancer tissues and adjacent non-cancerous tissues from patients who underwent surgery, and CAF-CM (conditioned medium) and NF-CM (conditioned medium) were prepared as described in our previous work 21. We chose the following NSCLC cell lines: A549 cells (adenocarcinoma), H1299 cells (carcinoma), and H661 cells (large cell carcinoma).

**Figure 1:**
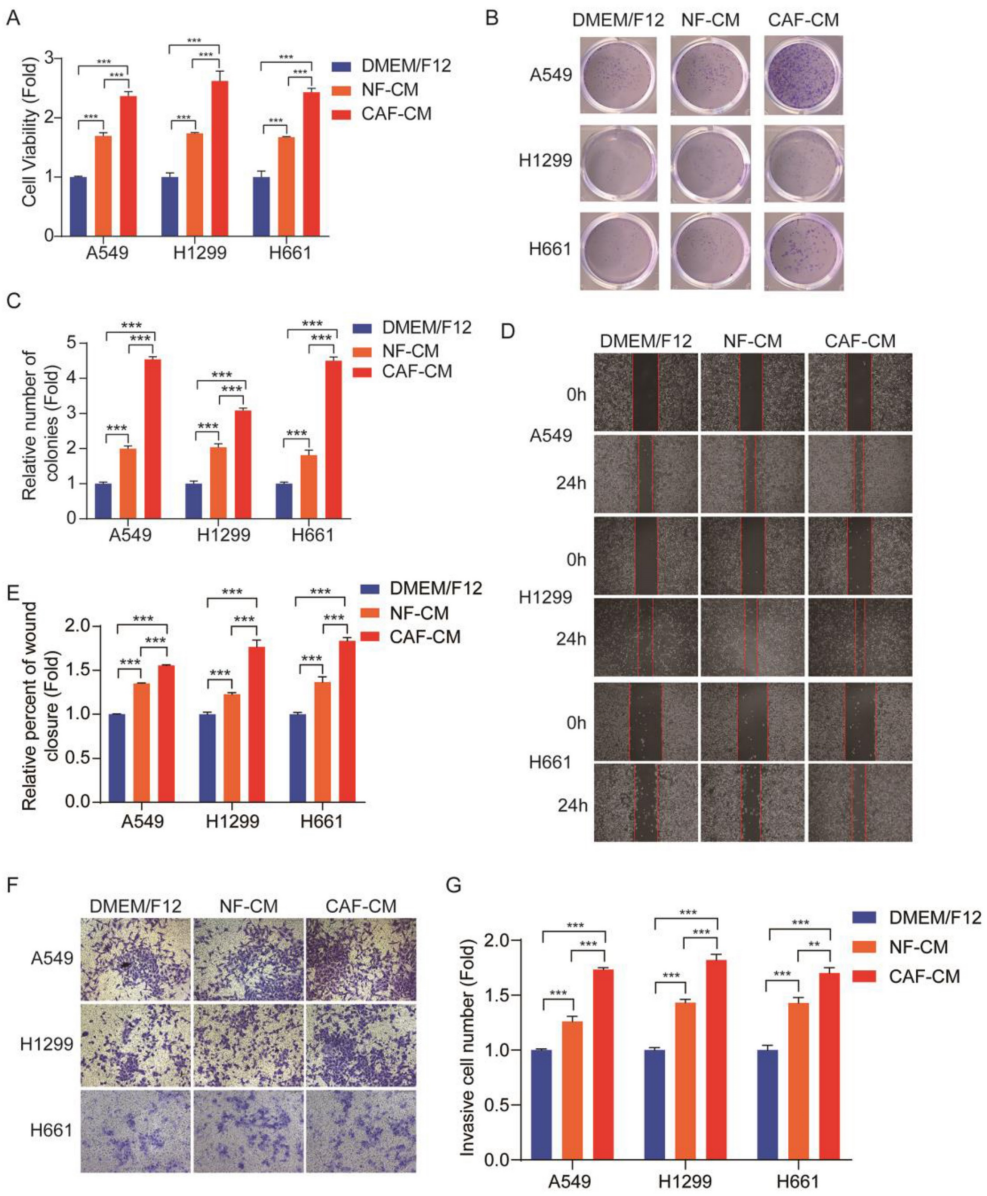
** CAFs facilitated growth, migration and invasion of NSCLC cells.** NSCLC cells were cultured in DMEM/F12 or NF-CM or CAF-CM. **(A)** Cell growth was detected by CCK-8 kit after 48 h. **(B, C)** The colony formation assay was performed after 1-2 weeks. **(D, E)** Cells were pre-treated with mitomycin C (0.05 μM) for 24 h. Cell migration was assessed by the wound healing assay after 24 h; representative photographs were presented (40×magnification). **(F, G)** Cells were pre-treated with mitomycin C (0.05 μM) for 24 h. Cell invasion was detected by transwell assay after 48 h, representative photographs were presented (100× magnification). Data represented the mean ± SD from three independent experiments. Columns, mean; bars, SD. *P<0.05, **P<0.01, ***P<0.001.

Cell proliferation was assessed by CCK-8 kit. Figure 1A shows that both CAFs and NFs enhanced NSCLC cells growth, and the effect of CAFs was stronger than that of NFs. The colony formation assay also indicated that CAFs promoted the colony-formation property of NSCLC cells (Figure 1B, C). The metastatic potential of tumor cells depends on their ability of migration and invasion. In order to investigate the migration and invasion of NSCLC cells, the wound healing assay and transwell assay were performed. Since CAFs enhanced cell proliferation, to exclude this effect on cell migration and invasion, the cells were pretreated with 0.05 μM mitomycin C, which inhibited cell proliferation, and around 85%-90% of the cells survived. The wound healing assay showed that both CAFs and NFs stimulated cell migration, but CAFs were more effective (Figure 1D, E). Similar results were also observed in the transwell assay (Figure 1F, G). Taken together, these results demonstrated that CAFs enhanced the growth and metastatic potential of NSCLC cells.

### CAFs increase m^6^A modification in NSCLC cells

**Figure 2:**
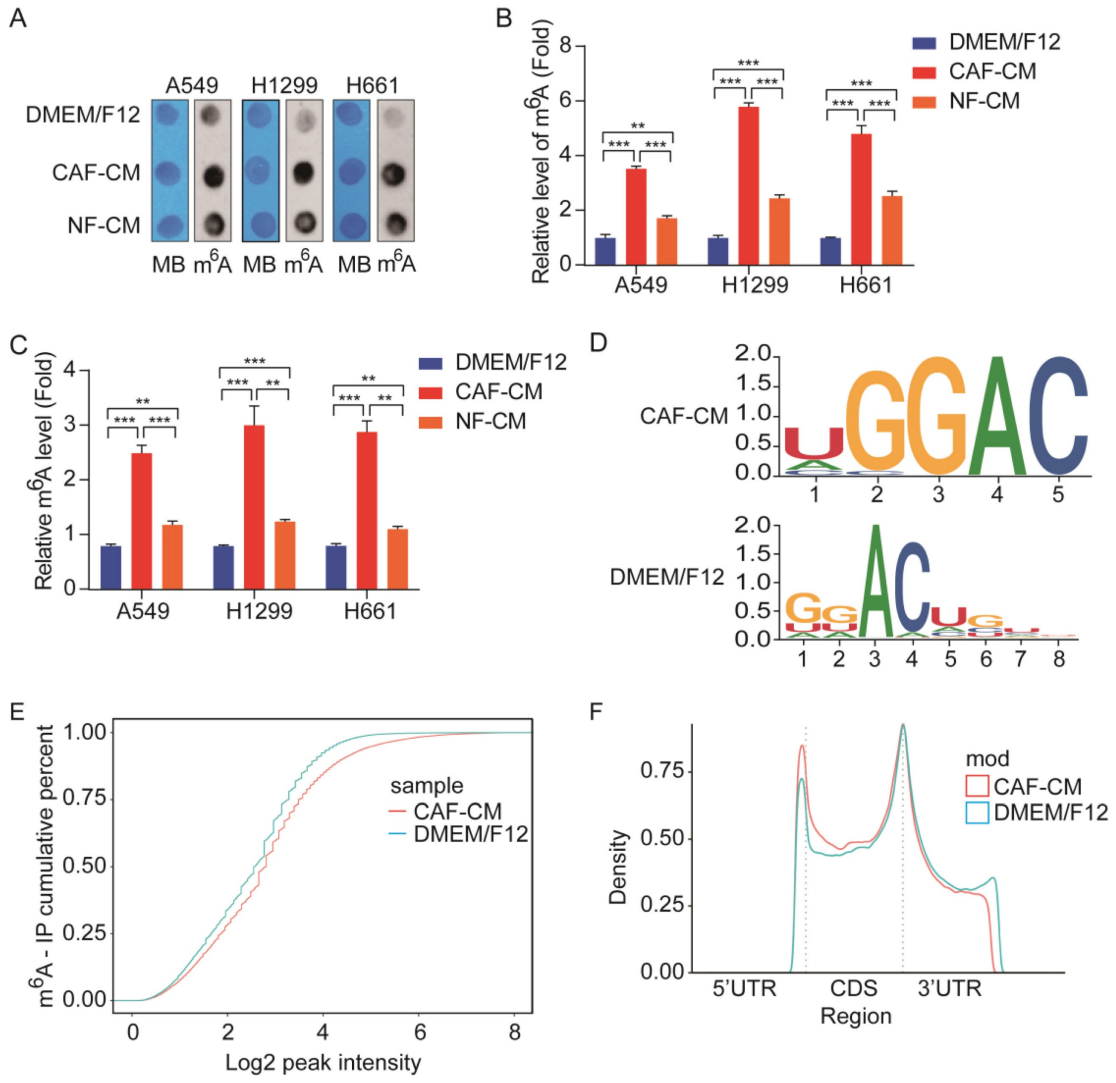
** CAFs elevated m^6^A modification in NSCLC cells.** A549 cells were cultured in DMEM/F12 or NF-CM or CAF-CM for 24 h. **(A, B)** m^6^A methylation was detected by RNA Dot Blot assay. Methylene blue (MB) staining served as a loading control. **(C)** The m^6^A methylation was detected by an m^6^A RNA Methylation Quantification Kit. **(D)** The m^6^A consensus sequence motif was identified by MeRIP-seq. **(E)** Comparison of m^6^A modification by cumulative distribution function (CDF curve). **(F)** Peak distribution of m^6^A modification in three segments, 5'UTR, CDS, and 3'UTR. Columns, mean; bars, SD. *P<0.05, **P<0.01, ***P<0.001.

Accumulating evidence suggests that aberrant m^6^A modification participates in cancer pathogenesis and progression 11; however, it is not clear whether CAFs affect m^6^A modification in lung cancer cells. To determine the effect of CAFs on m^6^A modification in lung cancer cells, NSCLC cells were treated with CAF-CM and NF-CM, and the levels of m^6^A modification in NSCLC cells were first detected by m^6^A RNA dot blot assay. As shown in Figure 2A and B, both CAFs and NFs elevated m^6^A modification levels in NSCLC cells, but CAFs were more effective. The m^6^A modification levels were also examined by m^6^A RNA Methylation Quantification Kit, and the results were in agreement with the dot blot assay results (Figure 2C).

We further performed MeRIP-seq on lung cancer cells exposed to CAFs. MeRIP-seq data analysis revealed that methylation on the m^6^A modification consensus 5'-RRACH-3' motif was significantly increased by CAFs (Figure 2D), which led to the elevation of general m^6^A level in CAFs-treated cells (Figure 2E). Further analysis indicated that m^6^A modifications were enriched in the coding sequence (CDS) region and 5'UTR region, and reduced in the 3'UTR region (Figure 2F). Collectively, the above results demonstrated that CAFs elevated m^6^A modifications in NSCLC cells.

### METTL3 mediates m^6^A modifications in NSCLC cells

**Figure 3:**
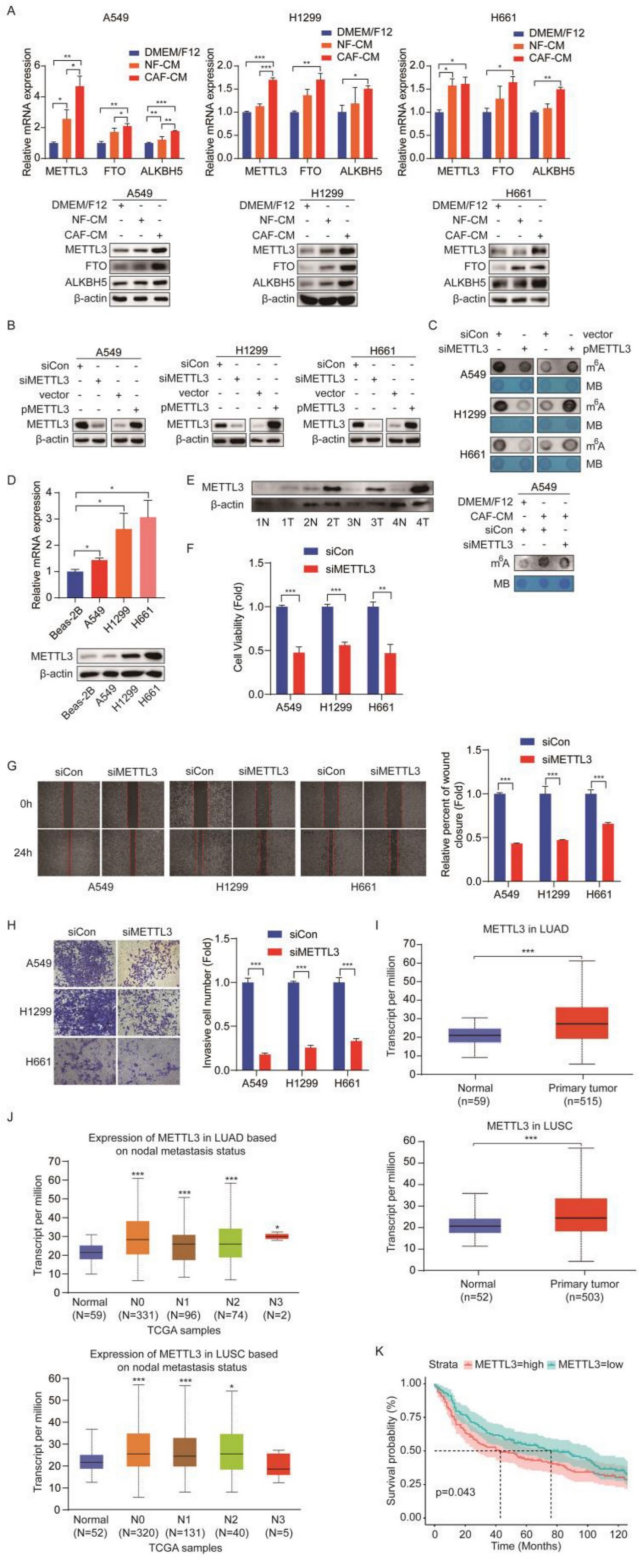
** METTL3 mediated the m^6^A methylation in NSCLC cells. (A)** The effect of CAFs on METTL3, FTO, ALKBH5 expression. **(B)** NSCLC cells were transfected with siRNA duplex or expression plasmid, and the efficiency of METTL3 knockdown and overexpression was detected by Western blotting. **(C)** M^6^A levels were detected by Dot Blot assay. MB staining served as a loading control. **(D)** The METTL3 expression was detected by qPCR and Western blotting in NSCLC cell lines.** (E)** The METTL3 expression was detected by Western blotting in paired lung cancer tumor and non-tumor specimens (n = 4). **(F)** METTL3 was knocked down and cell viability was examined by CCK-8 kit. **(G)** METTL3 was knocked down and cell migration was assessed by wounding healing assay.** (H)** METTL3 was knocked down and cell invasion were detected by transwell assay. **(I)** METTL3 levels in LUAD and LUSC patients from TCGA dataset. **(J)** The correlation between METTL3 levels and metastasis status. **(K)** Survival rate analysis of METTL3 levels in lung cancer patients (normal sample n = 14, tumor sample n = 293). Columns, mean; bars, SD. *P<0.05, **P<0.01, ***P<0.001.

Given that m^6^A modifications are catalyzed by m^6^A methyltransferases and removed by m^6^A demethylases, and CAFs increased m^6^A levels, we hypothesized that these m^6^A regulators were modulated by CAFs. To test our assumption, we detected the expression levels of methyltransferase METTL3 and demethylases FTO and ALKBH5 after CAFs treatment. Interestingly, we found that CAFs significantly increased the expression levels of all three regulators, at both mRNA level and protein level in NSCLC cells (Figure 3A). Since m^6^A modification level was elevated by CAFs, we therefore focused on methyltransferase METTL3, not demethylases FTO and ALKBH5. To determine the role of METTL3 in m^6^A modification in lung cancer cells, METTL3 was overexpressed or knocked down in lung cancer cells. The dot blot assay showed that m^6^A level increased when METTL3 was overexpressed, and decreased when METTL3 was knocked down, indicating that METTL3 introduced m^6^A modification into lung cancer cells (Figure 3B, C). We then treated METTL3-knockdown cells with CAF-CM. The results showed that METTL3-knockdown mitigated the enhancement of m^6^A modification by CAFs, suggesting that CAFs elevated m^6^A modification level through METTL3 (Figure 3C).

To further explore the role of METTL3 in lung cancer, we first compared the expression level of METTL3 in lung cancer cell lines with a human normal lung bronchial epithelium cell line (Beas-2B cells). As shown in Figure 3D, METTL3 was highly expressed in lung cancer cell lines. Importantly, when we compared the METTL3 level in paired tumor and non-tumor tissues, we found that METTL3 levels in the tumor tissues were significantly higher than those in the non-tumor tissues, suggesting that METTL3 might facilitate lung cancer progression (Figure 3E). To verify this hypothesis, METTL3 expression was reduced in lung cancer cells, and cell growth, migration, and invasion were measured. Compared with the control group, cell proliferation, migration, and invasion were dramatically inhibited by METTL3 knockdown (Figure 3F-H).

Based on the above findings in NSCLC cells, we next evaluated the clinical significance of METTL3 in NSCLC patients. We focused on lung adenocarcinoma (LUAD) and lung squamous cell carcinoma (LUSC), which are major subtypes of NSCLC. By analyzing the Cancer Genome Atlas (TCGA) database, we found that METTL3 was highly expressed in both LUAD and LUSC patients compared with healthy controls (Figure 3I). We next investigated the correlation between METTL3 level and nodal metastatic status. In LUAD, we found that METTL3 levels of tumor tissue at stage N0-N3 were significantly higher than those of normal tissue; similarly, in LUSC, METTL3 levels of tumor tissues at stage N0-N2 were significantly higher than those of normal tissue; however, there were no significant differences in METTL3 expression between stage N0 and N1-N2, suggesting that there was no correlation between METTL3 and nodal metastatic status (Figure 3J). Further GEO dataset (GSE30219 cohort, normal sample n = 14, tumor sample n = 293) analysis indicated that high METTL3 expression was associated with poor survival rate of patients with lung cancer (Figure 3K). Collectively, these results suggest that METTL3 is involved in NSCLC progression via the regulation of m^6^A modification.

### CAFs elevate METTL3 level in NSCLC cells via VEGFA secretion

**Figure 4:**
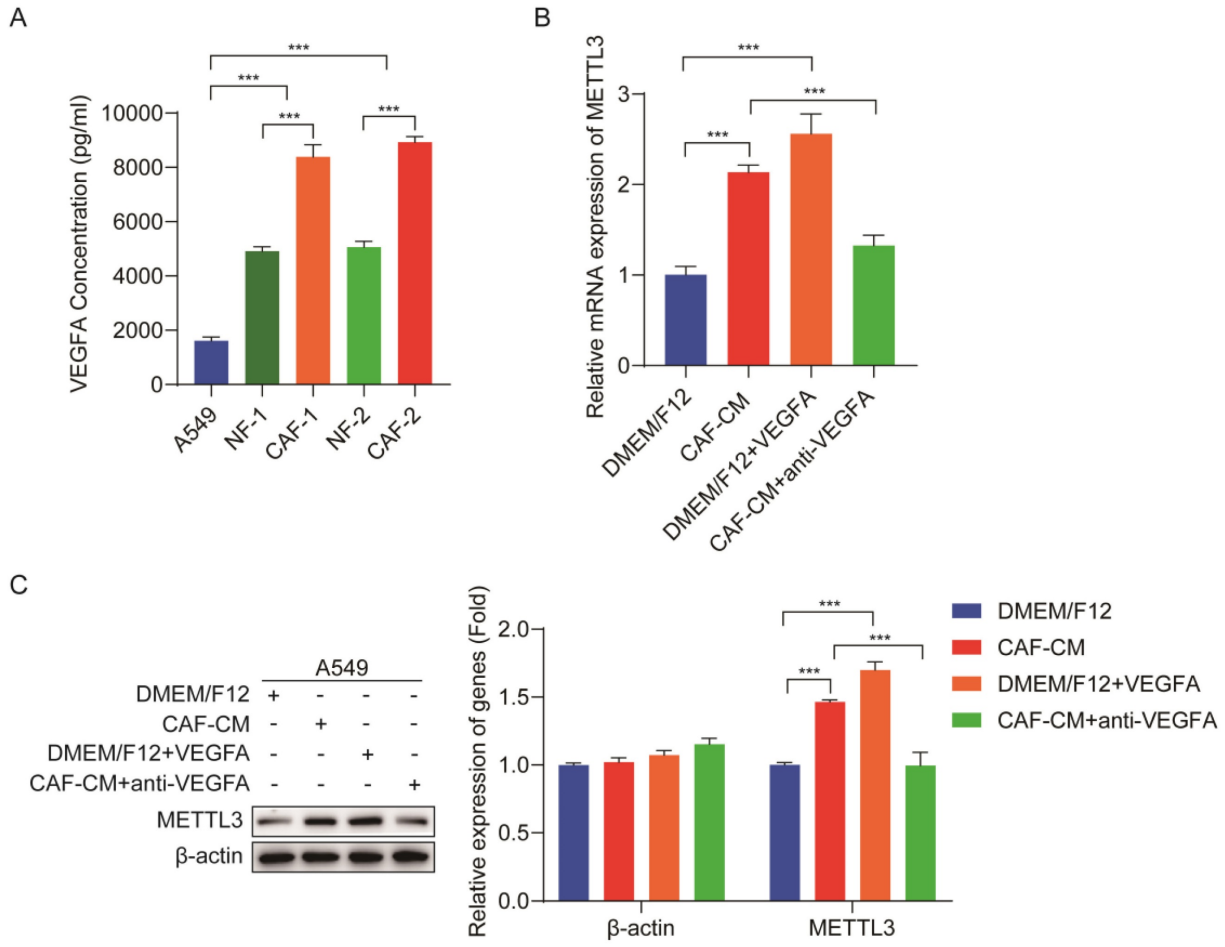
**CAFs elevated METTL3 level via VEGFA secretion. (A)** VEGFA secreted by A549 cells, NFs and CAFs were detected by ELISA kit.** (B)** Lung cancer cells were cultured with DMEM/F12, or CAF-CM, or DMEM/F12 + recombinant human VEGFA (20 ng/ml), or CAF-CM + VEGFA neutralizing antibody (5 μg/ml) for 48 h. METTL3 mRNA expression in lung cancer cells was detected by qPCR. **(C)** Protein expression of METTL3 in lung cancer cells was detected by Western blotting. Columns, mean; bars, SD. *P<0.05, **P<0.01, ***P<0.001.

Although we found that CAFs increased m^6^A level in NSCLC cells, it was not elucidated how CAFs regulate m^6^A level in NSCLC cells. VEGFA plays a crucial role in tumor progression 22; our previous study demonstrated that VEGFA secretion was responsible for the CAFs' effect on lung cancer metastasis 18. We therefore hypothesized that CAFs upregulated METTL3 in NSCLC cells via VEGFA secretion, and METTL3 upregulation led to m^6^A modification in NSCLC cells. To verify this assumption, we first detected the VEGFA level released from CAFs and lung cancer cells. ELISA results showed that both NFs and CAFs secreted higher amount of VEGFA than lung cancer cells, and the amount of VEGFA from CAFs was the highest (Figure 4A). To explore the effect of VEGFA on METTL3 expression in lung cancer cells, we next added recombinant VEGFA to DMEM/F12, or added VEGFA-neutralizing antibody to CAF-CM, and then used them to culture lung cancer cells. The expression levels of METTL3 in lung cancer cells were detected by qPCR and western blotting. As shown in Figure 4B, C, DMEM/F12 supplemented with recombinant VEGFA significantly increased METTL3 expression, which was similar to CAF-CM, whereas CAF-CM supplemented with VEGFA-neutralizing antibody dramatically mitigated the stimulating effect of CAF-CM on METTL3 expression. These results demonstrated that CAFs elevated METTL3 level in NSCLC cells via VEGFA secretion.

### CAFs increase m^6^A modification and expression of RAC3

**Figure 5:**
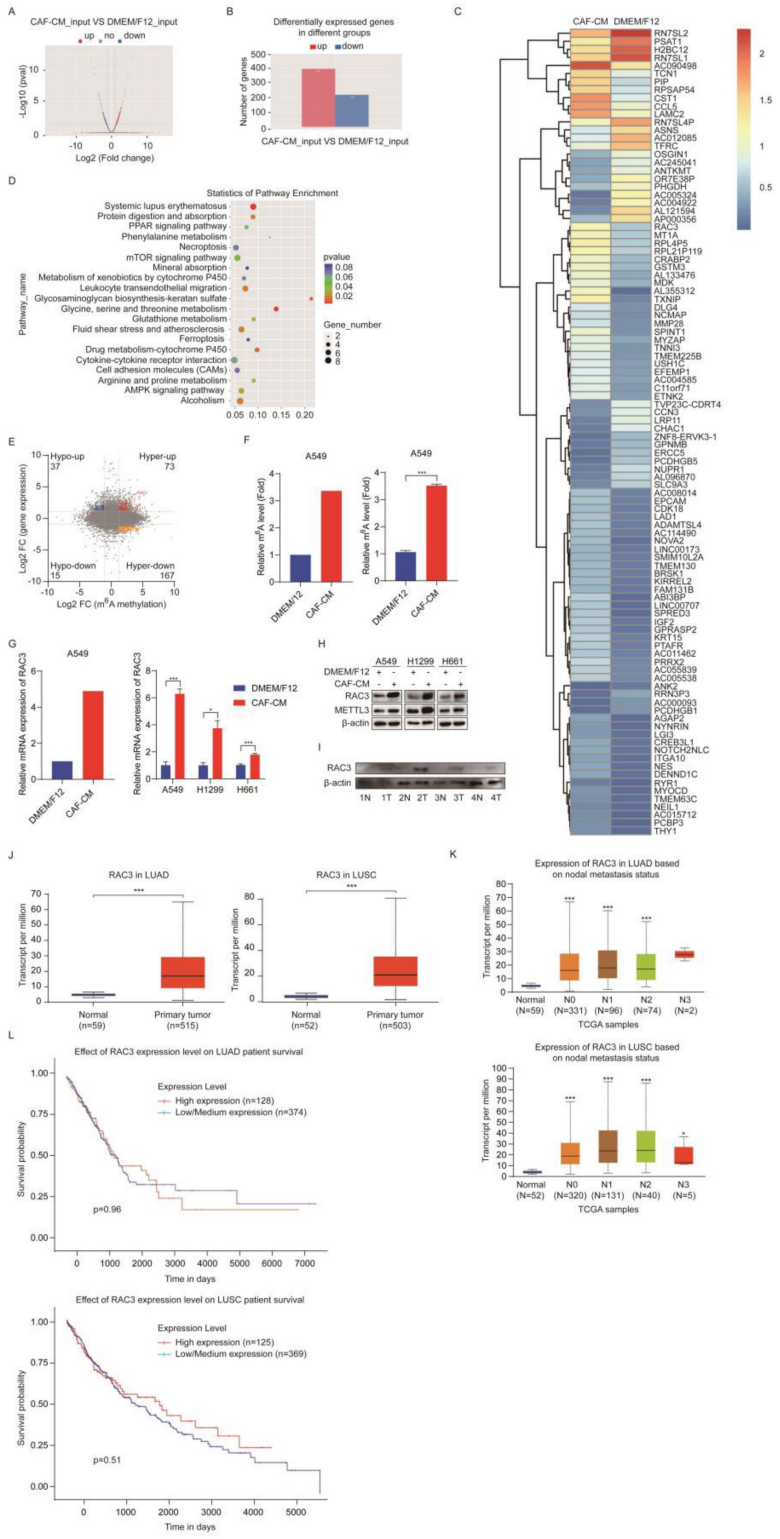
** CAFs increased m^6^A methylation and expression of RAC3. (A)** Volcano plot of mRNA levels detected by MeRIP-seq. Red dots meant high mRNA expression in CAF-CM cultured cells, while blue dots meant high mRNA expression in DMEM/F12 cultured cells. **(B)** Differentially expressed genes detected by RNA-seq. **(C)** Hierarchical clustering of genes differentially expressed. **(D)** KEGG pathway analysis of genes differentially expressed.** (E)** Distribution of peaks (fold change > 1.5 or < - 1.5, P < 0.05) with a significant change in mRNA level and m^6^A level in CAF-CM treatment *vs* DMEM/F12 treatment. **(F)** M^6^A modification of RAC3 mRNA detected by MeRIP-seq (left) or MeRIP-qPCR (right). **(G)** mRNA expression of RAC3 detected by RNA-seq (left) or qPCR (right).** (H)** The expressions of RAC3 and METTL3 were detected by Western blotting. **(I)** Expressions of RAC3 in paired lung tumor tissues and non-tumor tissues were detected by Western blotting. **(J)** RAC3 expression levels in LUAD and LUSC patients in the TCGA dataset. **(K)** Correlation between RAC3 expression levels and metastasis status.** (L)** Survival rate analysis of RAC3 expression level in lung cancer patients. Columns, mean; bars, SD. *P<0.05, **P<0.01, ***P<0.001.

m^6^A modification affects gene expression through regulating the translation, splicing, export, and degradation of RNA 11, which drove us to explore the target genes of METTL3. We performed transcriptomic RNA sequencing (RNA-seq) to investigate the mRNA expression. The RNA-seq data indicated that 397 genes were upregulated and 219 genes were downregulated in the CAFs-treated group (Figure 5A, B). The most dramatically regulated genes are listed in Figure 5C.

The KEGG pathway analysis revealed that the differentially expressed genes were enriched in the mTOR signaling pathway, AMPK signaling pathway, protein digestion and absorption pathway, and drug metabolism pathway (Figure 5D). We further investigated the genes changed at both mRNA level and m^6^A level by analyzing RNA-seq data combined with MeRIP-seq data. We found 73 hypermethylated m^6^A peaks with increased mRNA expression, and we called these peaks hyper-up genes. We also found 167 hyper-down genes, 37 hypo-up genes, and 15 hypo-down genes (Figure 5E).

Since m^6^A modification may increase gene expression, we next screened genes in the hyper-up group, particularly the genes that can stimulate cell growth. Among these genes, RAC3 drew our attention. In order to confirm the sequencing results, we further examined the m^6^A level of RAC3 by MeRIP-qPCR, and mRNA level of RAC3 by qPCR in the CAFs-treated samples. As shown in Figure 5F and G (left, sequencing results; right, qPCR results), both m^6^A level and mRNA level were increased, which were consistent with the MeRIP-seq and RNA-seq results. We also investigated the effect of CAFs on protein level of RAC3. The western blotting results indicated that RACs proteins were upregulated by CAFs in all three NSCLC cell lines, and METTL3 was also upregulated (Figure 5H). Moreover, we evaluated the clinical significance of RAC3 in lung cancer. As shown in Figure 5I, RAC3 level was higher in lung cancer specimens compared with the paired adjacent non-cancerous tissues. Further TCGA dataset analysis indicated that RAC3 was remarkably upregulated in both LUAD and LUSC tissues compared with non-cancerous tissues (Figure 5J). We further investigated the correlation between RAC3 level and nodal metastatic status. We found that RAC3 levels of tumor tissue at stage N0-N2 were higher than those of normal tissue in LUAD; similarly, RAC3 levels of tumor tissue at stage N0-N3 were significantly higher than those of normal tissue in LUSC; however, there were no significant differences in RAC3 levels of tumor tissue between stage N0 and N1-N2, suggesting that RAC3 did not correlate with nodal metastatic status (Figure 5K). Furthermore, RAC3 expression was not associated with prognosis (Figure 5L). Taken together, these results demonstrated that CAFs enhanced m^6^A modification and expression of RAC3, and RAC3 was not associated with NSCLC metastasis.

### METTL3 enhances m^6^A methylation and stability of RAC3 mRNA

**Figure 6:**
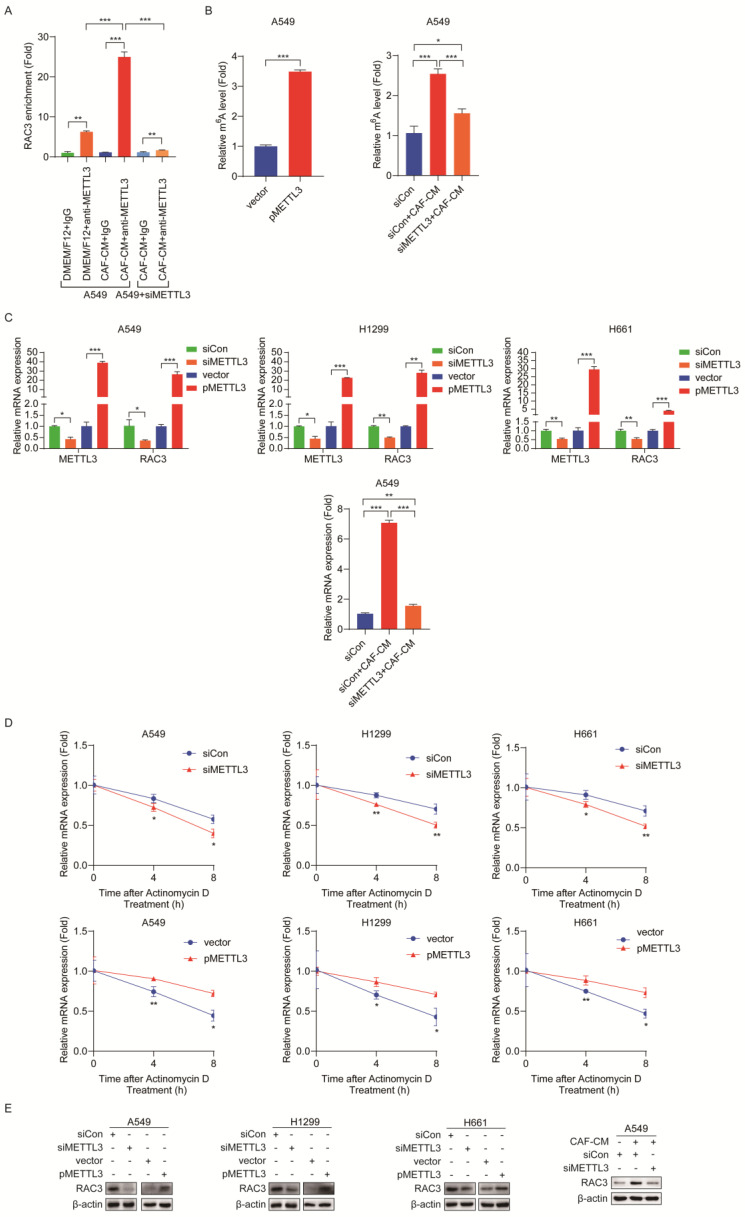
** METTL3 enhanced m^6^A methylation and stability of RAC3 mRNA. (A)** RIP-qPCR assay of RAC3 enrichment by METTL3 protein.** (B)** MeRIP-qPCR analysis was performed to determine the m^6^A level of RAC3 mRNA in A549 cells overexpressed METTL3 or reduced METTL3. **(C)** METTL3 was overexpressed or knocked down, respectively, and the mRNA levels of METTL3 and RAC3 were detected by qRT-PCR. **(D)** RNA stability of RAC3 mRNA in METTL3-overexpressing or knockdown NSCLC cells after treated with actinomycin D (5 μg/mL). **(E)** METTL3 was overexpressed or knocked down, RAC3 expression was detected by Western blotting. Columns, mean; bars, SD. *P<0.05, **P<0.01, ***P<0.001.

Given that CAFs increased m^6^A modification and expression of RAC3, we further explored whether the CAFs' effect on RAC3 mRNA was mediated by METTL3. To assess the direct interaction between METTL3 and RAC3 transcript, we performed RNA immunoprecipitation (RIP)-qPCR assays. As shown in Figure 6A, compared with the control antibody IgG, the METTL3-specific antibody significantly enriched RAC3 mRNA, and CAF-CM remarkably enhanced this binding. However, in METTL3-knockdown cells, the enhancement of the binding by CAFs was abrogated. The MeRIP-qPCR results further revealed that METTL3 overexpression dramatically increased m^6^A modification in RAC3 mRNA. Interestingly, when METTL3 was knocked down in lung cancer cells, the increase in m^6^A modification by CAFs was attenuated (Figure 6B).

Next, we investigated the effect of METTL3 on RAC3 transcription. As shown in Figure 6C, METTL3 knockdown decreased mRNA level of RAC3, whereas METTL3 overexpression increased RAC3 transcription. We further treated METTL3-knockdown cells with CAF-CM. As shown in Figure 6C, the stimulating effect of CAFs on RAC3 transcription was blocked when METTL3 was reduced.

Furthermore, we studied the effect of METTL3 on the stability of RAC3 mRNA by treating cells with actinomycin D. The RAC3 mRNA expression was initially decreased; interestingly, METTL3 silencing accelerated mRNA decay, while enforced METTL3 expression reversed these effects (Figure 6D). Moreover, at protein level, knockdown of METTL3 significantly reduced the protein level of RAC3; in contrast, overexpression of METTL3 significantly increased the protein level of RAC3 (Figure 6E). Notably, CAFs increased RAC3 expression; however, this effect was mitigated when METTL3 was knocked down (Figure 6E).

Collectively, our data indicated that METTL3 methylated RAC3 mRNA and maintained its stability by preventing degradation, which resulted in the increased RAC3 expression. CAFs increased RAC3 expression in a CAF-METTL3-m^6^A modification-dependent manner.

### CAFs enhance NSCLC cells migration via RAC3-mediated AKT/NF-κB pathway

**Figure 7:**
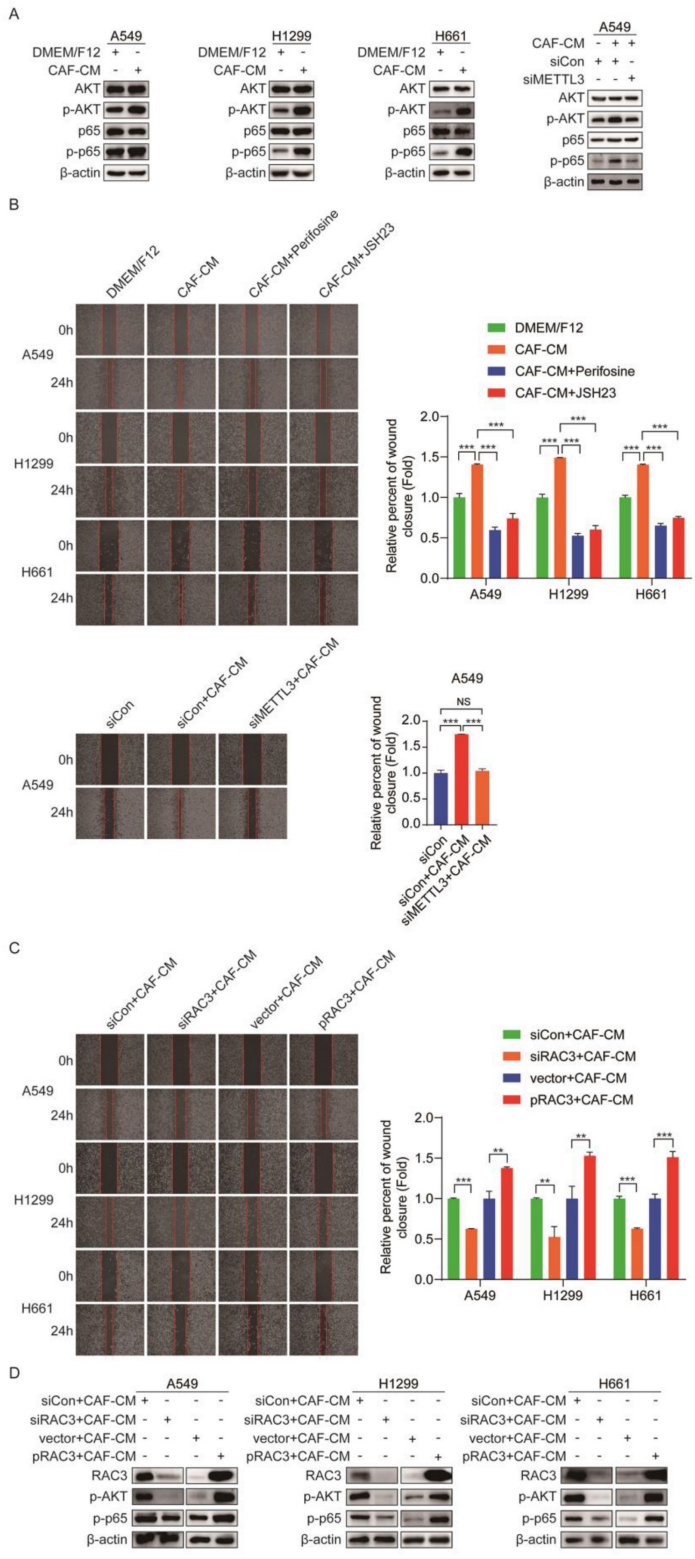
** CAFs enhanced NSCLC cells migration via RAC3 mediated AKT/NF-κB pathway. (A)** NSCLC cells were treated with CAF-CM. AKT/NF-κB signaling pathway was examined by Western blotting. **(B)** NSCLC cells were pre-treated with AKT inhibitor perifosine (10 μM) or NF-κB inhibitor JSH23 (14 μM) for 2 h, then treated with CAF-CM. Cell migration was detected by wound healing assay. **(C)** RAC3 was overexpressed or knocked down in NSCLC cells, AKT/NF-κB signaling pathway was examined by Western blotting.** (D)** RAC3 was knocked down in NSCLC cells. Cell migration was detected by wound healing assay. Columns, mean; bars, SD. *P<0.05, **P<0.01, ***P<0.001.

AKT and NF-κB are responsible for metastasis 23, 24. To explore the mechanism underlying the promoting role of CAFs in metastasis of NSCLC cells, we examined the effect of CAFs on the AKT/NF-κB pathway. As shown in Figure 7A, CAF-CM elevated the levels of p-AKT and p-p65, while the levels of total AKT and p65 remained unchanged. However, when METTL3 was knocked down, CAF-CM had no effect on the AKT/NF-κB pathway. Notably, when cells were pretreated with AKT inhibitor perifosine or NF-κB inhibitor JSH23, the enhancement effect of CAFs on cell migration was significantly mitigated (Figure 7B). Furthermore, we investigated the role of METTL3 in CAFs' effect on cell migration. As shown in Figure 7B, METTL3-knockdown attenuated the CAFs' promoting effect. These data suggested that CAFs promoted cell migration through the METTL3-mediated AKT/NF-κB pathway.

Numerous studies have shown that RAC3 is involved in tumor invasion and metastasis 25, 26, which drove us to further investigate whether RAC3 played a role in NSCLC cells migration. When RAC3 was knocked down in lung cancer cells, the enhanced migration by CAFs was diminished. On the contrary, when RAC3 was overexpressed in lung cancer cells, the enhanced migration by CAFs was boosted (Figure 7C). The effect of RAC3 on CAFs-activated AKT/NF-κB signaling was also evaluated. As shown in Figure 7D, RAC3 silencing mitigated the activation of the AKT/NF-κB pathway by CAFs, whereas RAC3 overexpression enhanced the activation of the AKT/NF-κB pathway. Given that CAFs upregulated RAC3 expression (Figure 5H), our data illustrated that CAFs enhanced NSCLC cells migration via RAC3-mediated AKT/NF-κB pathway activation.

### Inhibition of METTL3 attenuates CAFs' effect on lung cancer growth *in vivo*

**Figure 8:**
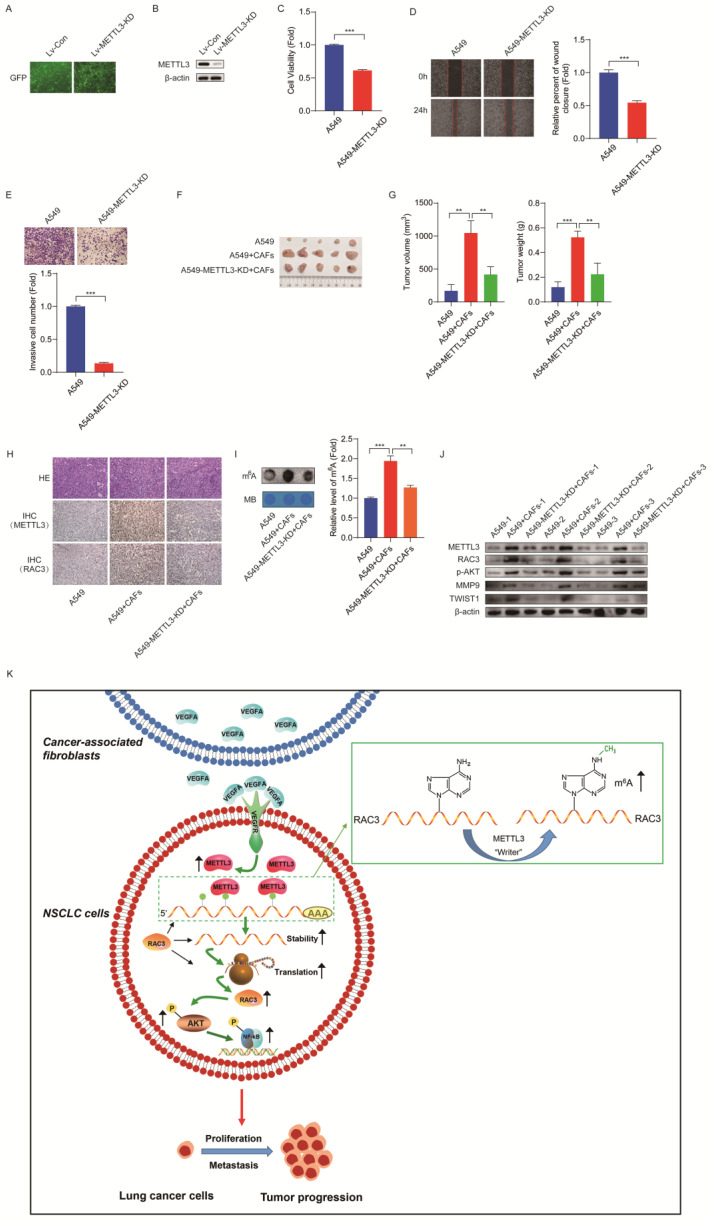
** Inhibition of METTL3 attenuated CAFs' effect on lung cancer growth *in vivo.* (A)** The efficiency of lentivirus infection was observed under fluorescence microscope by detecting GFP. **(B)** METTL3 knocked down was detected by Western blotting.** (C)** Cell growth was detected by CCK-8 kit after 48 h.** (D)** Cell migration was assessed by the wound healing assay after 24 h. **(E)** Cell invasion was detected by transwell assay after 48 h. **(F)** Mice were divided into 4 treatment groups including A549 cells, A549 cells + CAFs, A549-METTL3-KD cells, and A549-METTL3-KD cells + CAFs. Cells were subcutaneously injected into nude mice. (G) Tumor masses were collected after 5 weeks. The volumes and weights of tumor mass were measured. **(H)** Representative pictures of H&E staining, and IHC staining of METTL3 and RAC3 in tumor tissues (200 x). **(I)** m^6^A levels of tumor tissues were detected by dot blot assay. **(J)** The expressions of METTL3, RAC3, p-AKT and metastasis-related genes were detected by Western blotting. **(K)** Scheme illustrating CAFs' regulation on m^6^A modification in lung cancer cells. Columns, mean; bars, SD. *P<0.05, **P<0.01, ***P<0.001.

In order to evaluate the role of METTL3 in lung cancer growth *in vivo*, we first established a stable METTL3-knockdown cell line A549-METTL3-KD by lentivirus infection. The efficiency of lentivirus infection was examined by observing green fluorescence of GFP, and the efficiency of METTL3 knockdown was detected by western blotting (Figure 8A, B). The role of METTL3 in cell growth was assessed. As shown in Figure 8C-E, reduced METTL3 expression in lung cancer cells significantly suppressed cell growth, migration, and invasion.

We next clarified whether METTL3 affected the CAFs' effect on tumor growth* in vivo*. The mice were divided into four treatment groups, including A549 cells, A549 cells + CAFs, A549-METTL3-KD cells, and A549-METTL3-KD cells + CAFs. The cells were subcutaneously injected into nude mice. Tumor growth was determined by tumor volume and tumor weight. As shown in Figure 8F and G, CAFs dramatically stimulated tumor growth. Interestingly, we observed that A549-METTL3-KD cells did not form tumors. This is in line with our* in vitro* study, which indicated that METTL3 repression inhibited lung cancer cell growth. Notably, when METTL3 was knocked down in lung cancer cells, the stimulating effect of CAFs on tumor growth was significantly diminished.

Furthermore, we investigated the underlying mechanism using tumor tissues. Immunohistochemistry assay showed that CAFs increased the expression of METTL3 and RAC3 in tumor tissues (Figure 8H). The dot blot assay revealed that CAFs significantly elevated m^6^A level in tumor tissues; however, METTL3-knockdown abolished this effect (Figure 8I). Moreover, CAFs upregulated metastasis-related genes MMP9 and Twist1, upregulated RAC3, and activated AKT (Figure 8J). These findings were in agreement with our *in vitro* study, suggesting that CAFs promoted lung cancer growth through the METTL3-mediated m^6^A modification of RAC3 mRNA, and activated metastasis-related genes and the AKT pathway.

## Discussion

Metastases are one of the leading causes of cancer-related death. While for many years, cancer research has focused on tumor cells, recent studies have been concentrating on the communication between tumor cells and TME. As one of the major components of TME, CAFs have been shown to play a critical role in metastasis in various types of cancer. CAFs enhance ovarian cancer invasion and metastasis through DDR2-regulated periostin 27 and promote colorectal cancer metastasis through IL-6-mediated LRG1 upregulation 28. Li YX* et al.* reported that ACLP activated CAFs to promote metastasis of pancreatic cancer by regulating the expression of MMP1 and MMP3 in CAFs 29. Our previous studies also revealed that CAFs facilitated lung cancer metastasis by secreting IL-6, KRT8, HMGB1, and VEGFA 8, 9, 18, 21. However, the mechanism underlying the CAFs' promoting effect is still not fully understood.

mRNA modification is a new layer of posttranscriptional gene regulation. Among different mRNA modifications, m^6^A modification is the most abundant form. m^6^A modification may regulate mRNA fate, including processing, decay, and translation. Recent studies have revealed that m^6^A modification is involved in lung cancer progression. VIRMA promoted NSCLC progression through DAPK3 mRNA degradation via VIRMA-guided m^6^A modification 30. lncRNA AC098934 enhanced the invasion of lung adenocarcinoma cells through METTL3-mediated m^6^A modification in AC098934 31. ALKBH5 inhibited TGF-β/SMAD signaling and suppressed TGF-β-induced epithelial-mesenchymal transition (EMT) through reducing m^6^A modifications in NSCLC cells 32. However, whether m^6^A modification is responsible for the CAFs' effect on lung cancer metastasis has not been evaluated. In the present study, we found that CAFs promoted NSCLC cell migration and invasion by elevating the m^6^A level in NSCLC cells.

m^6^A modification is a reversible dynamic process regulated by WERs (“writers,” “erasers,” and “readers”). As one of the writers, METTL3 installs m^6^A modification, and plays an important role in lung cancer. METTL3 is responsible for TGF-β-induced EMT in lung cancer cells through regulating JUNB 33. METTL3 upregulated Bcl-2 via m^6^A modification, leading to enhanced growth and migration of NSCLC cells 34. Xue L *et al.* showed that METTL3 mediated m^6^A modification in lncRNA ABHD11-AS1 and increased lncRNA ABHD11-AS1 expression, which promoted the Warburg effect of NSCLC 35. In line with these findings, we found that METTL3-regulated m^6^A methylation mediated the promoting effect of CAFs on NSCLC cell migration and invasion. Notably, when METTL3 expression was repressed, the promoting effect of CAFs on NSCLC cells migration was dramatically mitigated. Further* in vivo* study also demonstrated that METTL3 inhibition abolished the CAFs' stimulatory effect on tumor growth, suggesting that METTL3 could be a potential therapeutic target for NSCLC treatment. Furthermore, our analysis of multiple clinical datasets revealed that METTL3 is associated with NSCLC prognosis, providing evidence for METTL3 as a promising biomarker for prognosis of NSCLC.

Rho family of small guanosine triphosphatases (GTPases) plays a crucial role in many pathological processes; it consists of three members, including RAC1, RAC2, and RAC3. There is increasing evidence that RAC3 is involved in tumor progression 36. RAC3 is highly expressed in bladder cancer, and is associated with pathological grades and stages; in addition, RAC3 upregulation correlates with poor prognosis 37. RAC3 facilitates proliferation and invasion of bladder cancer cells via PYCR1/JAK/STAT signaling 26, and promotes invasion and metastasis of breast cancer cells through modulating adhesion and matrix degradation 38. In lung cancer, RAC3 accelerates cell proliferation by regulating cell cycle 39, and stimulates EMT and cell invasion through the p38 MAPK pathway 40. In this study, we demonstrated that METTL3 directly regulated m^6^A modification of RAC3 mRNA, increased RAC3 transcription, mRNA stability and translation. Our results also showed that RAC3 knockdown abrogated the CAFs' stimulatory effect on cell migration. Notably, TCGA database analysis indicated that RAC3 was upregulated in both LUAD and LUSC tissues compared with non-tumor tissues, suggesting the potential of RAC3 as a therapeutic target.

It has been reported that RAC3 acts as an oncogene, regulating cell functions through diverse downstream signaling pathways. RAC3 is highly expressed in bladder cancer cells, and stimulates proliferation and migration of bladder cancer cells via PYCR1-mediated JAK/STAT pathway activation 26. Interestingly, when RAC3 was knocked down, the growth and migration of bladder cancer cells were inhibited through the PI3K/AKT/mTOR pathway-mediated autophagy induction 25. RAC3 inhibition also caused a significant reduction of both invasion and adhesion of breast cancer cells through the ERK-2/NF-κB signaling pathway 41. In lung cancer, Zhang *et al.* reported that RAC3 regulated EMT and migration of lung cancer cells through the p38 MAPK pathway 40. However, it has not been evaluated whether RAC3 activates the AKT/NF-κB pathway in lung cancer cells. Here, we revealed that RAC3 activated the AKT/NF-κB pathway, and RAC3-mediated AKT/NF-κB pathway activation was responsible for CAFs' effect on NSCLC cell migration.

In summary, CAFs promote the metastatic potential of NSCLC cells through an m^6^A modification-dependent regulatory mechanism, and METTL3 mediates m^6^A modification in NSCLC cells. We further identified RAC3 as a downstream target of METTL3, and RAC3 facilitates NSCLC cell migration via the AKT/NF-κB pathway, suggesting that RAC3 m^6^A modification may serve as a potential therapeutic target for lung cancer treatment (Figure 8K). “Readers” recognize and bind to m^6^A methylated sites to control mRNA decay and translation. However, it has not been evaluated whether “readers” are involved in RAC3 regulation. It is worthy of further investigation whether “readers” might be responsible for RAC3 expression.

## Figures and Tables

**Table 1:** PCR primer sequences.

Primers	Sequence (5′-3′)		Length of amplicons (bp)
METTL3	Forward	CAAGGAGGAGTGCATGAAAG	212
	Reverse	GGCTTGGCGTGTGGTCTTTG	
RAC3	Forward	TCCCCACCGTTTTTGACAACT	187
	Reverse	GCACGAACATTCTCGAAGGAG	
FTO	Forward	GACTCTCATCTCGAAGGCAG	220
	Reverse	CCAAGGTTCCTGTTGAGCAC	
ALKBH5	Forward	GTTCCAGTTCAAGCCTATTC	260
	Reverse	GGTCCCTGTTGTTTCCTGAC	
VEGFA	Forward	AGGGCAGAATCATCACGAAGT	75
	Reverse	AGGGTCTCGATTGGATGGCA	
GAPDH	Forward	TGCACCACCAACTGCTTAGC	87
	Reverse	GGCATGGACTGTGGTCATGAG	

METTL3: methyltransferase like3; RAC3: Rac family small GTPase 3; FTO: fat mass and obesity associated; ALKBH5: alkylation repair homolog 5; VEGFA: vascular endothelial growth factor A; GAPDH, Glyceraldehyde-3-phosphate dehydrogenase.

## Abbreviations

TME: Tumor microenvironment
CAFs: Cancer-associated fibroblasts
NSCLC: Non-small cell lung cancer
m^6^A: N6-methyladenosine
METTL3: Methyltransferase-like 3
qPCR: Quantitative PCR
MeRIP-qPCR: Methylated RNA immunoprecipitation quantitative PCR
MeRIP-seq: Methylated RNA immunoprecipitation sequencing
IHC: Immunohistochemistry
EMT: epithelial-mesenchymal transition
